# MEPs and MRI Motor Band Sign as Potential Complementary Markers of Upper Motor Neuron Involvement in Amyotrophic Lateral Sclerosis

**DOI:** 10.1111/ene.70055

**Published:** 2025-02-06

**Authors:** Francesca Calvi, Andrea Fortuna, Luca Bello, Mariagiulia Anglani, Diego Cecchin, Daniele Sabbatini, Cinzia Andrigo, Marcello Ferullo, Susanna Ruggero, Marco Falda, Elena Pegoraro, Gianni Sorarù

**Affiliations:** ^1^ Department of Neurosciences, Motor Neuron Disease Center University Hospital of Padova Padua Italy; ^2^ Neuroradiology Unit University Hospital of Padova Padua Italy; ^3^ Nuclear Medicine Unit University Hospital of Padova Padua Italy; ^4^ Unit of Biostatistics, Epidemiology and Public Health, Department of Cardiac, Thoracic, Vascular Sciences, and Public Health University of Padova Padova Italy; ^5^ Motor Neuron Disease Center, Department of Neurosciences University Hospital of Padova Padua Italy

**Keywords:** ALS, PET‐MR, PUMNS, upper motor neuron

## Abstract

**Background:**

Amyotrophic lateral sclerosis (ALS) is characterized by the degeneration of both upper and lower motor neurons (UMNs and LMNs). Recognizing the involvement of UMNs is challenging because of the absence of reliable biomarkers beyond clinical evaluation.

**Aim:**

To identify a reliable marker of UMN damage in a cohort of patients with ALS referring to the Motor Neuron Disease Clinic of the University Hospital of Padova.

**Methods:**

We retrospectively evaluated the clinical records of 79 patients with ALS and compared the results of various investigations, including the motor‐evoked potentials (MEPs), positron emission tomography–magnetic resonance imaging (MRI) and light neurofilaments (NfLs), with the degree of UMN clinical involvement, as assessed by the Penn Upper Motor Neuron Score (PUMNS).

**Results:**

MEPs, considering the central motor conduction time (CMCT) values in both the upper and lower limbs, showed a significant correlation with the relative PUMNS subscores (*p* = 0.01, *ρ* = 0.4; and *p* = 0.005, *ρ* = 0.45, respectively). Additionally, there was a positive correlation between NfLs and PUMNS values (*p* = 0.04, *ρ* = 0.33). The presence of the motor band sign on MRI was associated with higher PUMNS values. Receiver operating characteristic analysis revealed that PUMNS accurately predicted abnormalities in CMCT values (specificity 86%, sensitivity 62%) and the presence of the motor band sign (specificity 58%, sensitivity 80%).

**Interpretation:**

In our cohort of patients with ALS, CMCT values proved to be the most reliable test for assessing UMN involvement, albeit the presence of the motor band sign on MRI showed higher sensitivity.

## 1 Introduction

Amyotrophic lateral sclerosis (ALS) is a devastating neurodegenerative disorder characterized by progressive degeneration of both upper and lower motor neurons (UMNs and LMNs, respectively), leading to muscle weakness and atrophy. Death usually occurs within 3 years after symptom onset because of respiratory failure [1, 2].

The diagnosis of ALS mainly relies on documented progressive symptoms and signs of both UMN and LMN dysfunction, along with investigations that exclude other pathological processes explaining motor neuron involvement [3].

UMN dysfunction is clinically characterized by the presence of one or more of the following: hyperreflexia with pathological reflex spread, spasticity, clonus, preserved reflexes in weak and wasted limbs, and the Babinski or Hoffmann signs [4]. However, confirming UMN clinical deficits may be challenging as they can be masked by concurrent LMN degeneration, especially in the early stages of the disease [4]. Objective markers for UMN involvement are crucial for diagnosis, as syndromes solely affecting LMNs may stem not only from the ALS spectrum but also from mimics such as motor neuropathies, Kennedy's disease, and adult‐onset spinal muscular atrophy [5]. Notably, post‐mortem examinations have shown corticospinal tract (CST) degeneration in up to 75% of patients diagnosed with progressive muscular atrophy (PMA), the ALS subtype characterized by clinically pure LMN involvement [6].

Assessing UMN burden requires a thorough clinical evaluation and precise quantification of UMN findings. Employing reliable and standardized methods for clinical assessment is essential to guarantee the accuracy and consistency of data. The Penn Upper Motor Neuron Score (PUMNS) serves as a standardized measure of UMN signs in ALS. PUMNS is not correlated with age or disease duration. Past research has shown that increased scores on this tool correspond to diffusion tensor imaging metrics indicative of disease progression and more extensive pathology in the CST [7].

According to the latest revision of the ALS diagnostic criteria, known as the Gold Coast criteria [3], supportive evidence of UMN dysfunction can be derived from transcranial magnetic stimulation (TMS) studies, magnetic resonance imaging (MRI), and neurofilament (NF) levels.

TMS enables non‐invasive assessment of the functional integrity of the motor cortex and its corticomotoneuronal projections. TMS‐induced motor evoked potentials (MEPs) serve as a non‐invasive means of evaluating damage to the central motor pathways in ALS [8]. The central motor conduction time (CMCT), which measures the time it takes for stimuli to reach the bulbar or spinal motoneurons following motor cortex stimulation, is regularly documented as an indicator of UMN damage in clinical practice [9, 10, 11].

Neuroimaging techniques can objectively visualize changes associated with the disease process and aid in understanding the mechanisms of neurodegeneration in vivo. In ALS, conventional MRI is currently used primarily to exclude disease mimics rather than to confirm the diagnosis [12]. However, it can reveal hyperintense signaling on T2‐weighted or T2‐fluid attenuation inversion recovery (FLAIR) images involving the CSTs. Additionally, a hypointensity of the precentral gyrus, known as the motor band sign, likely related to iron deposition, can also be detected in T2‐weighted images and, more prominently, in susceptibility‐weighted images (SWI) [13, 14]. MRI may be complemented by positron emission tomography (PET), which provides a unique opportunity to acquire both MRI and PET information in a single imaging session, thus enhancing the mapping of vulnerable brain regions [15]. Clinical studies using 2‐deoxy‐2‐18fluoro‐D‐glucose‐PET, also referred to as [18F]FDG PET, have provided compelling evidence of glucose hypometabolism in the motor cortices of patients with ALS [16, 17].

NFs are a structural component of the axonal cytoskeleton and are released into cerebrospinal fluid (CSF) and peripheral blood as a consequence of axonal damage [18]. Among neurodegenerative diseases, NF levels tend to be highest in ALS [19], where both light and heavy‐chain NFs (NfLs and NfHs, respectively) are considered diagnostic and prognostic biomarkers [19, 20]. In ALS, UMN degeneration plays a significant role in the release of NFs into biofluids [20, 21, 22, 23]. However, the relative contribution of LMN axonal destruction may also be important [21, 23].

With the goal of establishing a reliable marker of UMN damage beyond clinical evaluation, we compared the results of CSF NfLs, CMCT, and PET‐MRI investigations with the degree of UMN clinical involvement, as assessed by PUMNS values, in a cohort of patients with ALS referring to our Motor Neuron Disease (MND) Clinic.

## 2 Methods

### 2.1 Patients

We conducted a retrospective evaluation of clinical records from 79 patients diagnosed with ALS [24] who were consecutively hospitalized for diagnostic purposes at the in‐patient service of the MND Clinic of the University Hospital of Padova between January 2016 and September 2023. The study considered the following demographic and clinical characteristics collected during hospitalization: sex; age at onset and admission to our MND Clinic; and revised ALS Functional Rating Scale score (ALSFRS‐R) [25]. Additionally, we recorded results of the following studies conducted during hospitalization:

1. PUMNS assessment: This 28‐item semi‐quantitative scale evaluates UMN burden with total scores ranging from 0 to 32 (0–4 for the bulbar region, and 0–7 for each limb). For certain analyses, we also considered the upper and lower limb subscores (ranging from 0 to 14 each) of the scale. Higher score values correspond to a greater UMN burden [7].
2. MEPs: We used a “MagPro Compact” magnetic stimulator from Medtronic. The CST to the upper limbs was recorded from the abductor digiti minimi muscle, and to the lower limbs from the abductor hallucis muscle. The CMCT was calculated with the indirect method (using F‐wave). Values exceeding 15 ms for the lower limbs and 8 ms for the upper limbs, based on the mean plus 2.0 standard deviations of a healthy control group (consisting of 70 subjects matched for sex and age), were considered abnormal. In cases where a reliable CMCT was hindered because of severe UMN pathway alterations, an arbitrary value of 25 m per second (m/s) for the lower limbs and 20 m/s for the upper limbs was assigned.
3. Brain PET/MRI: Adhering to the guidelines of the European Association of Nuclear Medicine, [26] patients underwent a 6‐h fasting period before the scan, and blood glucose levels were ensured to be less than 200 mg/dL. A single intravenous bolus of 3 MBq/kg of 18F‐FDG was administered 1 h before the scan. Simultaneous acquisition of PET/MRI scans was performed using a Siemens Healthcare Biograph mMR. The MRI component included T1‐ and T2‐weighted, T2‐FLAIR, SWI, and diffusion‐weighted image scans of the brain (3 T main magnetic field, 256 × 256 mm matrix, 1.00 mm thickness, TR = 2400 ms, TE = 3.24 ms). Additionally, a PET brain scan was acquired with an acquisition time of 1500 s, a 344 × 344 mm matrix, and a 3D iterative reconstruction method. A neuroradiologist (MA) and a nuclear medicine specialist (DC), both possessing extensive experience with brain PET/MRI images, conducted examinations to identify specific features of interest: the motor band sign in the SWI sequence and hyperintensity of the CST bundles in the FLAIR sequence, each categorized as “present” or “not present”; and degree of hypometabolism at the motor cortex level, classified as absent, mild to moderate, or marked based on visual inspection.
4. NfLs: CSF samples were obtained through lumbar puncture, then centrifuged, aliquoted, and stored at −80°C. Standard CSF analysis was conducted within 1–2 h after sample collection. NfLs were measured using an NfL enzyme‐linked immunosorbent assay (UmanDiagnostics AB) [92]. According to our laboratory reference range, we considered pathological values as follows: > 560 pg/mL for patients aged 30–40 years, > 890 pg/mL for patients aged 40–60 years, and > 1850 pg/mL for patients older than 60 years.

This study was performed in accordance with the principles of the Declaration of Helsinki. Approval was granted by the Comitato Etico per la Sperimentazione Clinica della Provincia di Padova.

### 2.2 Statistic Analysis

PUMNS values were treated as a nonparametric variable and were therefore compared within different groups (PET‐MRI and MEPs) using the Wilcoxon–Mann–Whitney test. For qualitative variables, the chi‐squared test was employed. Correlations between quantitative variables (PUMNS and NfL, PUMNS, or CMCT) were assessed using the Spearman correlation coefficient. Receiver operating characteristic (ROC) analysis was conducted to evaluate PUMNS accuracy in anticipating abnormalities of MEPs, PET‐MRI, NfLs, or their combinations. The area under the ROC curve (AUC) was utilized to compare the performance of the different tests. Statistical significance was set at *p* < 0.05.

## 3 Results

The study cohort consisted of 79 patients, comprising 44 males and 35 females, with a median age of symptom onset at 60 years (range 35–81) and 63 at hospitalization (range 35–84). The median disease duration at the time of hospitalization was 14 months (range 6–92). The median ALSFRS‐R score was 42 (range 26–47).

In Table S1, we summarized the characteristics of the patients and also detailed, for each investigation considered, the number of patients who participated in each assessment.

**FIGURE 1:**
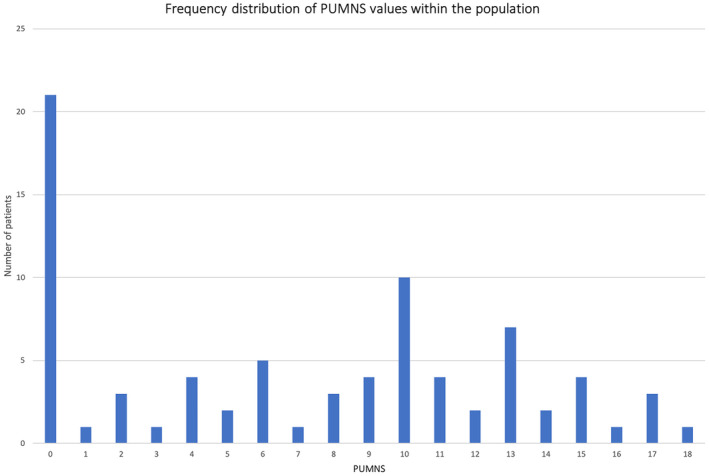
Frequency distribution of PUMNS values within the studied population. In our cohort, no patients exhibited a PUMNS score exceeding 18 (PUMNS score range, 0–32).

PUMNS was obtained for all patients and ranged from 0 to 18, with a median value of 8. None of the patients had a score above 18 (PUMNS score range, 0–32). The frequency distribution of PUMNS within the cohort is shown in Figure 1.

### 3.1 PUMNS Values and NfLs

**FIGURE 2:**
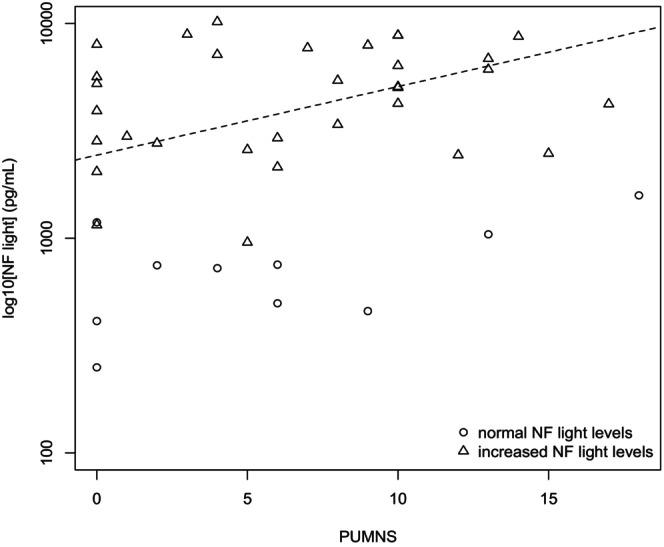
Correlation analysis of CSF Log[NfL] concentrations with PUMNS values. Correlations between quantitative variables (PUMNS and NfL) were assessed using the Spearman correlation coefficient (*ρ* = 0.33, *p* = 0.04). Triangles indicate increased NF light levels; circles indicate normal NF light levels; the dashed line represents the regression line calculated using the ordinary least squares (OLS) method considering all values.

NfL concentrations were determined in the CSF of 73 patients and ranged from 250.05 to 32,072.00 pg/mL. In 58 patients, NfL levels were found to exceed the age‐adjusted reference range. We observed a statistically significant positive correlation between NfLs and PUMNS values (*p* = 0.04, *ρ* = 0.33) (Figure 2). Furthermore, as anticipated [20], there was a significant inverse correlation between ALSFRS‐R scores and NfLs (*p* = 0.0085, *ρ* = −0.306), indicating that higher NfL levels were associated with lower ALSFRS‐R scores.

### 3.2 PUMNS Values and CMCT

**FIGURE 3:**
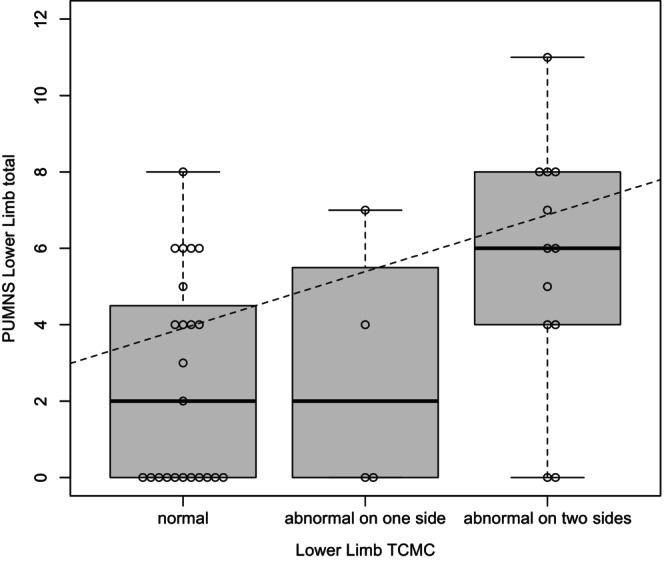
Box plots depicting the distribution of PUMNS lower limb subscore across different levels of CMCT values categorized as normal, abnormal on one side, or abnormal on two sides. ‘Abnormal on one side’ indicates that at least one of the values (right or left) is altered, whereas ‘abnormal on two sides’ signifies that both values (right and left) are altered. The dashed trend lines represent the regression line generated using the OLS method, providing a visual indication of the positive correlation between the increase in the PUMNS lower limb total score and different levels of the CMCT. The Spearman correlation coefficient was used to determine the strength and direction of the correlation (*p* = 0.01, *ρ* = 0.4). The median and interquartile range are shown.

**FIGURE 4:**
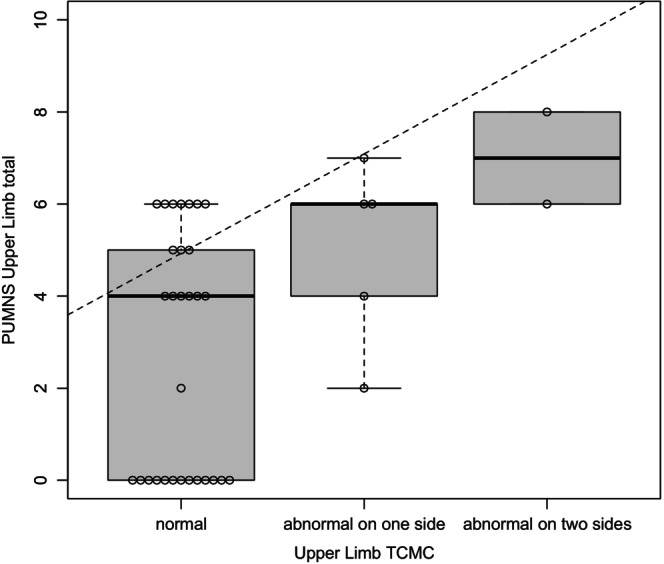
Box plots depicting the distribution of PUMNS upper limb subscore across different levels of CMCT values categorized as normal, abnormal on one side, or abnormal on two sides. ‘Abnormal on one side’ indicates that at least one of the values (right or left) is altered, whereas ‘abnormal on two sides’ signifies that both values (right and left) are altered. The dashed trend lines represent the regression line generated using the ordinary least squares method, providing a visual indication of the positive correlation between the increase in the PUMNS upper limb subscore and different levels of the CMCT. The Spearman correlation coefficient was used to determine the strength and direction of the correlation (*p* = 0.005, *ρ* = 0.45). The median and interquartile range are shown.

MEP studies were conducted in 39 patients, with assessments limited to upper limbs in two cases. For both upper and lower limbs, we examined the association between CMCT values and the corresponding PUMNS subscores. The statistical analysis was conducted categorizing patients into the following distinct groups on the basis of CMCT values: (1) normal CMCT values (23 and 30 patients for the lower and upper limbs, respectively); (2) increased CMCT values on one side (four and five patients for the lower and upper limbs, respectively); and (3) increased CMCT values bilaterally (12 and two patients for the lower and upper limbs, respectively). Using this classification, we found a significant correlation between PUMNS subscores and abnormal CMCT at both the lower and upper limb levels (*p* = 0.01, *ρ* = 0.4; and *p* = 0.005, *ρ* = 0.45, respectively) (Figures 3 and 4). Among patients with PUMNS = 0, four patients (10.2%) exhibited abnormal CMCT values in at least one limb.

A significant correlation was noted when comparing individual PUMNS values with the corresponding CMCT values obtained for each limb (Figure S1).

### 3.3 PUMNS Values and Brain [18F]FDG‐PET‐MRI

**FIGURE 5:**
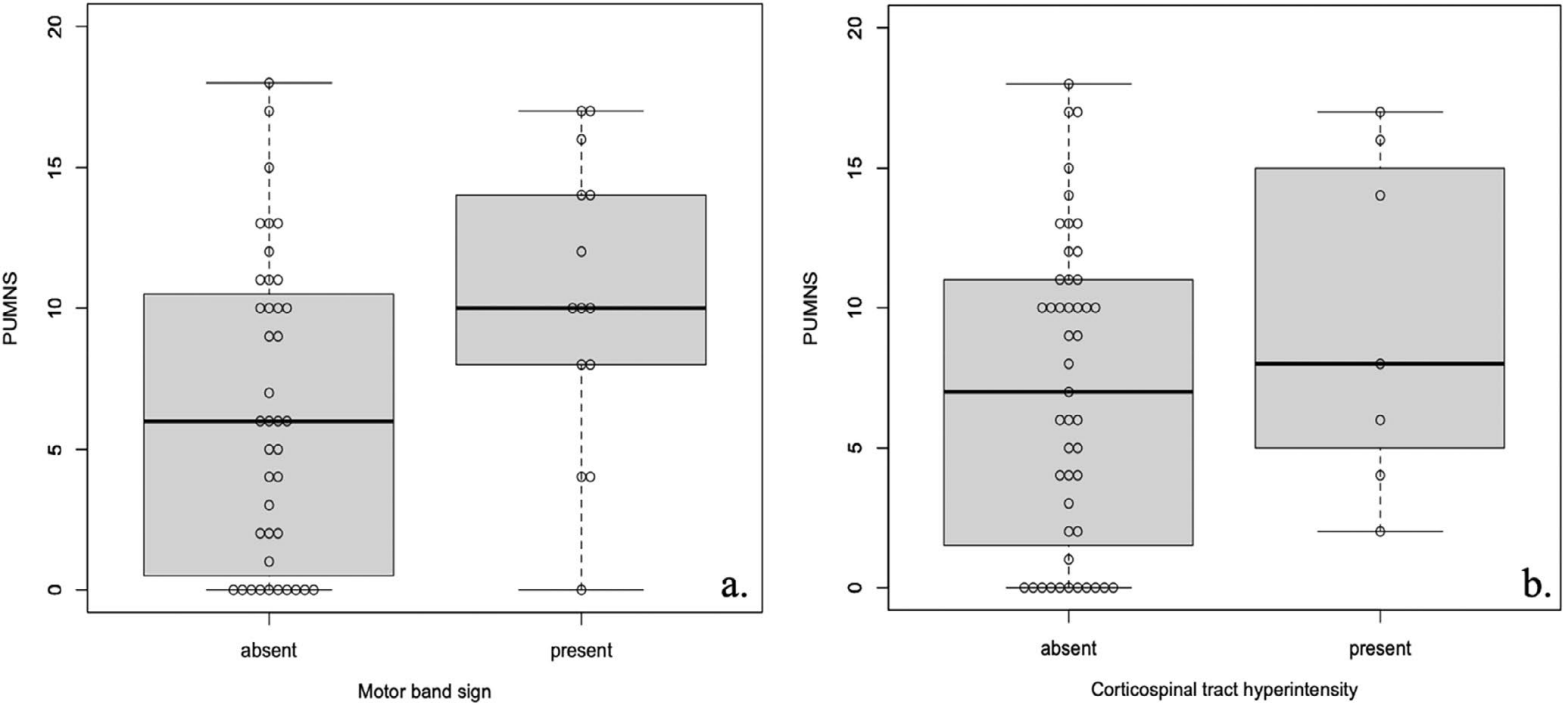
Box plots illustrating the relationship between PUMNS and the presence or absence of the motor band sign (a) and corticospinal tract (CST) hyperintensity (b). The bold line indicates the median, the box represents the interquartile range, and the whiskers show the minimum and maximum values. Patients with the motor band sign on at least one side had significantly higher PUMNS (*p* = 0.015), whereas no significant difference was observed for those with CST hyperintensity (*p* = 0.144). The Wilcoxon–Mann–Whitney test was used to assess data distribution.

Sixty patients underwent brain [18F]FDG‐PET‐MRI scans. MRI evaluation revealed the presence of the motor band sign in 15 patients (bilateral in 8 cases) and CST hyperintensity in seven patients (bilateral in 4 cases). Higher PUMNS values were observed in patients exhibiting the motor band sign on at least one side (*p* = 0.015) (Figure 5a), whereas no significant difference was observed in those with CST hyperintensity (*p* = 0.144) (Figure 5b). One patient with a PUMNS of 0 showed MRI abnormalities.

**FIGURE 6:**
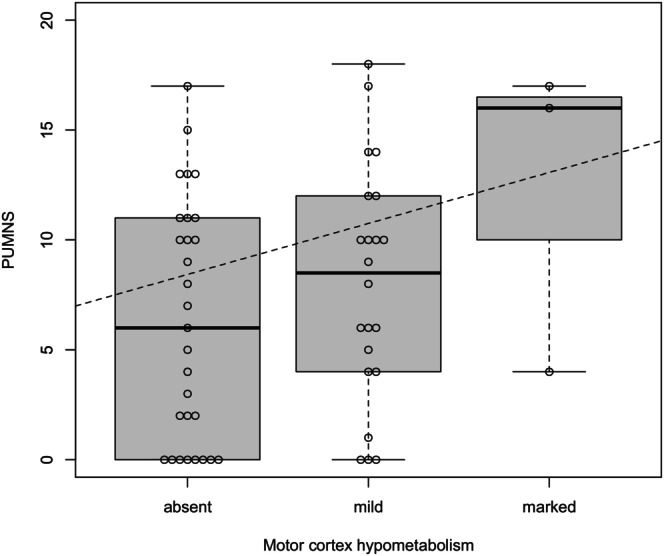
Box plots illustrating the correlation between PUMNS scores and varying degrees of hypometabolism in the motor cortex. The dashed trend lines represent the regression line generated using the OLS method. No significant difference was observed between hypometabolism levels and PUMNS scores (*p* = 0.9). The Spearman correlation coefficient was used to evaluate the strength and direction of the correlation. The median and interquartile range are shown.

According to the PET study, motor cortex hypometabolism was observed in 28 patients; it was mild in 25 cases (bilateral in 13) and marked in three patients (bilateral in 2 cases). No significant difference was found between the degree of hypometabolism and PUMNS values (*p* = 0.9), although there appeared to be a trend of increasing hypometabolism with higher PUMNS values (Figure 6). Three patients with a PUMNS of 0 showed mild hypometabolism of the motor cortex.

One notable observation is that among patients with normal motor cortex metabolism (32 out of 33 cases), the motor band sign was consistently absent. In contrast, among those with mild hypometabolism (25 cases), the motor band sign was present in nearly half of the cases (11 cases). Furthermore, in patients with marked hypometabolism (3 cases), the motor band sign was present in all cases.

### 3.4 ROC Analysis

**FIGURE 7:**
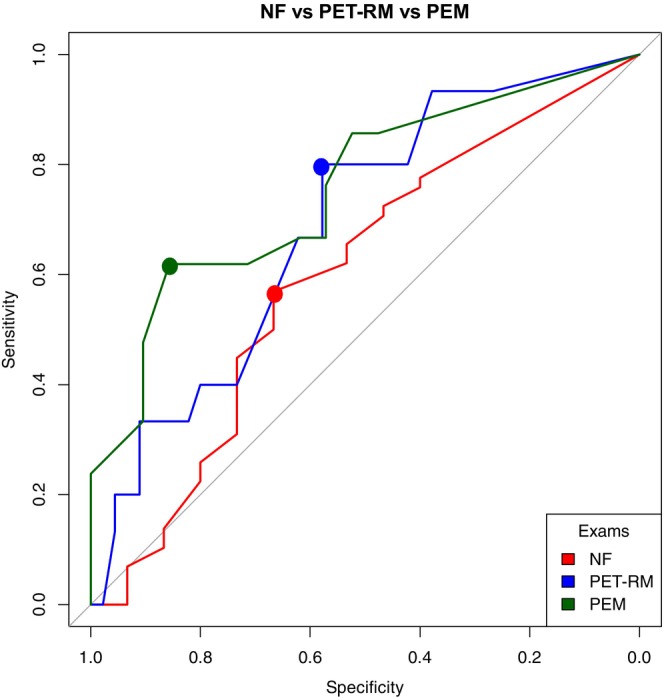
AUC values for the individual tests: NfL, PET‐MRI, and MEPs.

We employed ROC analysis to assess the predictive accuracy of PUMNS for abnormalities in NfLs, MEPs, or PET‐MRI. NfL levels were categorized as normal or abnormal on the basis of age‐adjusted reference ranges. Pathological conditions for MEPs were defined by the presence of at least one delayed CMCT value, regardless of the limb. Considering the correlation study findings from PET‐MRI studies, we included only the presence or absence of the motor band sign on at least one side as a parameter for statistical analysis. The outcomes of the individual ROC analyses for each investigation are depicted in Figure 7. Briefly, significant results were found for both MEPs (AUC = 0.857, *p* = 0.002 with a PUMNS threshold value of 9.5, specificity 86%, sensitivity 62%) and the presence of the motor band sign (AUC = 0.688, *p* = 0.0148; threshold = 7.5, specificity 58%, sensitivity 80%). The AUC for NfL levels was 0.58 (*p* = 0.122), with a threshold of 6.5 (specificity 67%, sensitivity 57%).

We subsequently performed ROC analysis on the aggregated study findings. The AUC for NfLs combined with PET‐MRI (54 patients) was 0.591 (*p* = 0.197; threshold = 9.5, specificity 78%, sensitivity 47%), and for NfLs combined with MEPs (36 patients), it was 0.744 (*p* = 0.030; threshold = 5.0, specificity 83%, sensitivity 67%). Notably, in the latter subgroup of patients, the individual AUCs for NfL and MEPs were as follows:

1. NfL: AUC = 0.669 (*p* = 0.059), with a threshold of 5 (specificity 70%, sensitivity 70%);
2. MEPs: AUC = 0.733 (*p* = 0.0079), with two thresholds identified: 9.5 (specificity 89%, sensitivity 56%) and 10.5 (specificity 95%, sensitivity 50%).

Twenty‐three patients underwent both PET‐MRI and MEPs. The AUC for these tests combined was 0.714 (*p* = 0.056), with a threshold of 3 (specificity 57%, sensitivity 87%).

Finally, results of all three investigations were available for 17 patients (AUC = 0.714, *p* = 0.141; threshold = 3, specificity 67%, sensitivity 86%). Full data are summarized in Figure S2 and Table S1.

## 4 Discussion

The diagnosis of ALS relies on identifying documented progressive symptoms and signs associated with dysfunction in either UMNs or LMNs [3]. However, accurately recognizing UMN involvement remains challenging, and the absence of a reliable biomarker for UMN damage beyond clinical evidence represents a significant issue [3].

This study aimed to explore the extent to which findings from CSF NfLs, brain PET‐MRI, and MEPs reflect UMN involvement, quantified by PUMNS, in patients with ALS. The findings suggest that MEPs, particularly CMCT values, may be the most reliable test, albeit the presence of the motor band sign on MRI showed higher sensitivity.

MEP study enables a non‐invasive assessment of UMN integrity and, importantly, is readily accessible in clinical practice [27]. In our study, we included CMCT measurement, which is known to be more frequently delayed in patients with UMN signs, [Ingram] reflecting axonal degeneration of the fastest conducting corticomotoneuronal fibers [27]. We observed a correlation between PUMNS values and the degree of abnormal CMCT distribution across all four limbs, and between individual PUMNS values and the corresponding CMCT values at each limb level. Furthermore, ROC analysis suggested that CMCT abnormalities may be accurately predicted by the burden of UMN involvement according to PUMNS, with high specificity but poor sensitivity. Therefore, MEPs seem to be a reliable surrogate marker of UMN involvement, particularly for ruling it out. Unlike our findings, where abnormal CMCT was detected in a minority of patients (10%) with PUMNS = 0, Zoccolella et al. [28] reported altered MEPs in over 70% of patients without clinical evidence of UMN involvement within their cohort. Given that both cohorts included patients in an early disease stage, this disparity might be partly due to the limitation of our MEP analysis being confined to CMCT studies and partly due to the high variability of the CMCT that is known to occur in patients with ALS [29, 30].

PET‐MRI allowed us to simultaneously investigate the structural and functional involvement of motor cortical and corticospinal dysfunction. MRI‐based results indicated that the motor band sign on at least one side is most prevalent in patients with higher PUMNS values, although with weak statistical significance. Additionally, an increase in PUMNS appeared to predict the presence of the motor band sign with good sensitivity (80%) but low specificity (58%). These findings align with the existing literature reporting a correlation with the degree of UMN impairment [31]. The low specificity echoes the observation that hypointensities in the precentral gyrus have also been described in healthy aging controls [31]. Unlike the motor band sign, PUMNS values were unrelated to the presence of the CST hyperintensity. CST hyperintensity has been actually reported to poorly correlate with UMN signs, [5] although this finding may be biased by the bulbar onset, which favors UMN degeneration and spread [31] and was not taken into account in our study. Regarding brain PET imaging, we examined the presence of motor cortex hypometabolism; however, we observed only a trend towards decreasing hypometabolism with increasing PUMNS values. Three patients without UMN signs displayed mild hypometabolism, possibly underscoring subclinical corticospinal damage in these cases. Similar results were previously observed in patients diagnosed with PMA [32]. In patients with ALS, other brain regions may exhibit hypometabolism or even hypermetabolism on [18F]FDG‐PET; [32, 33] however, we focused solely on the motor cortex as part of the UMN pathway.

A weak significant positive correlation between NfLs and PUMNS was noted. However, the highest NfL values were observed in patients with the classical ALS phenotype (i.e., with UMN and LMN signs), and NfL levels inversely correlated with the ALSFRS‐R score, regardless of PUMNS values. Additionally, the ROC curve for NfLs did not reach statistical significance. Overall, these findings suggest that NfLs may not be an exclusive or reliable marker of UMN involvement, consistent with previous reports [23, 34].

To increase the likelihood of objectively demonstrating UMN dysfunction, we conducted ROC analysis on the aggregated study findings. We omitted CST hyperintensity and PET data because of their lack of correlation with PUMNS values. We observed a significant association with PUMNS only when the combination included MEP results, thus providing further support for CMCT abnormalities as a reliable marker for UMN impairment.

In conclusion, our findings suggest that MEP studies may be the most effective paraclinical investigation for assessing UMN involvement in patients with ALS. We considered only CMCT abnormalities, but several other measures may be obtained through TMS techniques [27] to improve the diagnostic accuracy of neurophysiological studies. MEPs also have the advantage of being readily accessible in clinical practice. At the same time, the presence of the motor band sign on MRI emerged from our analysis as a highly sensitive test, and combining MEPs with PET‐MRI (for the motor band sign) increased the sensitivity up to 87%. Thus, a suggestion might be that both tests should be performed to prove UMN dysfunction.

Our study has several limitations that reduce the power of the results, including a small sample size, incomplete availability of certain investigations for all patients, and the restriction of MEP analysis to CMCT measurement. Regarding the incomplete availability of investigations, it is important to emphasize the retrospective design of the study, which inherently limits the ability to collect complete protocol data. Additionally, as our MND Centre is a tertiary referral center, many patients were referred to us after having already undergone some of the investigations considered in this study. In many cases, patients refused to repeat tests, particularly invasive ones, that they had already undergone.

Overall, these limitations highlight the need for larger‐scale, more comprehensive studies to validate and further substantiate our findings.

## Author Contributions

**Francesca Calvi:** investigation, writing – original draft, methodology, data curation, conceptualization, formal analysis. **Andrea Fortuna:** investigation. **Luca Bello:** formal analysis, supervision, project administration. **Mariagiulia Anglani:** data curation, validation. **Diego Cecchin:** validation, data curation. **Daniele Sabbatini:** formal analysis. **Cinzia Andrigo:** supervision. **Marcello Ferullo:** data curation, methodology. **Susanna Ruggero:** data curation. **Marco Falda:** formal analysis, data curation. **Elena Pegoraro:** supervision. **Gianni Sorarù:** writing – review and editing, project administration, supervision, methodology, investigation, resources, validation.

## Conflicts of Interest

The authors declare no conflicts of interest.

## Supporting information


Data S1.

## Data Availability

The datasets used and/or analyzed during the current study are available from the corresponding author on reasonable request.
