# Supplementary material for: MEPs and MRI Motor Band Sign as Potential Complementary Markers of Upper Motor Neuron Involvement in Amyotrophic Lateral Sclerosis
Source: Eur J Neurol. 2025 Feb 6;32(2):e70055. doi: 10.1111/ene.70055 (PMC11802645; doi:10.1111/ene.70055)
Supplement: Supplementary file 1 — Data S1. [file ENE-32-e70055-s001.docx]

**SUPPLEMENTARY**

Table S1: summary of selected characteristics of the study population, including age, sex and total Penn Upper Motor Neuron Score (PUMNS). The table also shows the number of patients who underwent neurofilament (NfL) testing, Motor Evoked Potentials (MEPs) testing in the upper and lower limbs, and PET-MRI. A checkmark (✓) indicates patients who completed each examination, while an 'X' indicates those who did not. The total number of patients per test is provided at the bottom of the table.

p-value = 0.037, ρ = 0.33

p-value = 0.005, ρ = 0.44

p-value = 0.0077, ρ = 0.43

p-value = 0.017, ρ = 0.41

Figure S1: Correlation between PUMNS subscores and CMCT for each individual limb


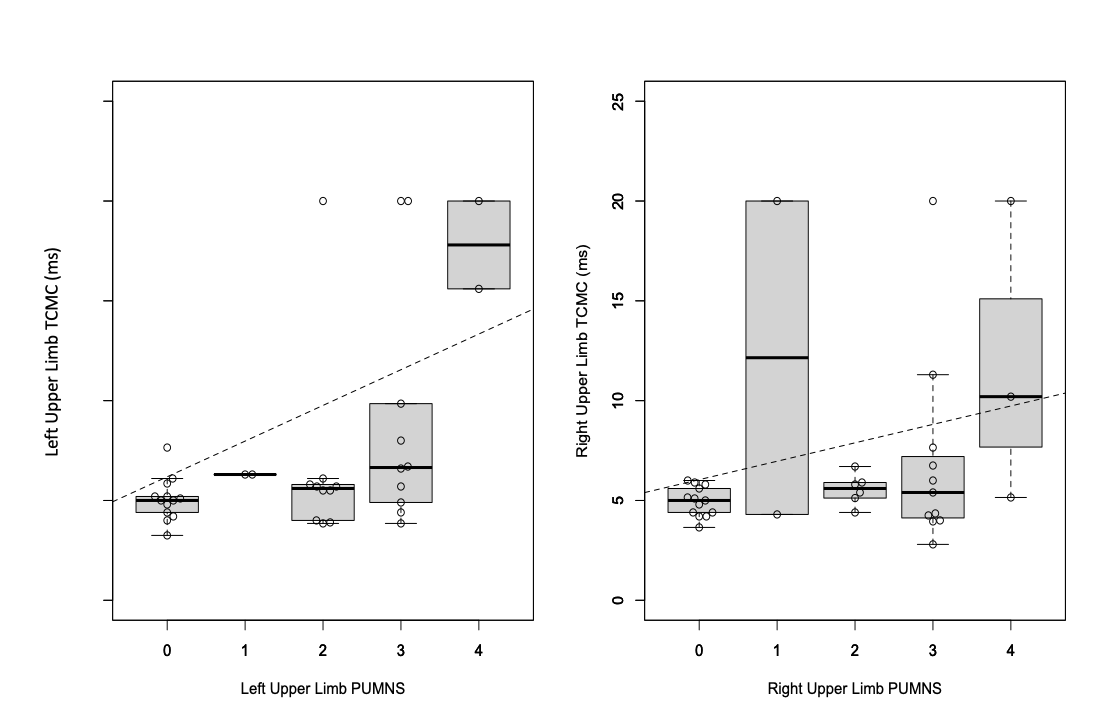

Table S2: results of ROC analysis. The results of combined tests are reported on rows without square brackets, while the result of the single test, conducted within the subset of the population that underwent those tests, is reported on rows with square brackets. The result refers to the test not enclosed within the square brackets.

Figure S2: results of ROC analysis for combined tests. Each graph displays the AUC of the combined tests and the AUC of single tests, considering only the population that underwent those tests.
